# Rapid-cycle evaluation and learning for the effective delivery of integrated interventions in early childhood in rural India

**DOI:** 10.3389/fpubh.2023.1013005

**Published:** 2023-02-03

**Authors:** Abhay Gaidhane, Shital Telrandhe, Manoj Patil, Penny A. Holding, Mahalaqua Nazli Khatib, Shilpa Gaidhane, Zahiruddin Quazi Syed, Sonali G. Choudhari, Roshan Umate, Aniket Pathade

**Affiliations:** ^1^Centre of One Health, Department of Community Medicine, School of Epidemiology and Public Health, Jawaharlal Nehru Medical College, Datta Meghe Institute of Higher Education and Research, Wardha, Maharashtra, India; ^2^Centre of Early Childhood Development – Stepping Stones Project, Department of Research and Development, Datta Meghe Institute of Higher Education and Research, Wardha, Maharashtra, India; ^3^i-Health Consortium, School of Epidemiology and Public Health, Jawaharlal Nehru Medical College, Datta Meghe Institute of Higher Education and Research, Wardha, Maharashtra, India; ^4^School of Epidemiology and Public Health, Jawaharlal Nehru Medical College, Datta Meghe Institute of Higher Education and Research, Wardha, Maharashtra, India; ^5^Global Evidence Synthesis Initiative, Division of Evidence Synthesis, Department of Physiology, Jawaharlal Nehru Medical College, Datta Meghe Institute of Higher Education and Research, Wardha, Maharashtra, India; ^6^Department of Medicine, Jawaharlal Nehru Medical College, Datta Meghe Institute of Higher Education and Research, Wardha, Maharashtra, India; ^7^Department of Clinical Epidemiology, Jawaharlal Nehru Medical College, Datta Meghe Institute of Higher Education and Research, Wardha, Maharashtra, India; ^8^Department of Community Medicine, Jawaharlal Nehru Medical College, Datta Meghe Institute of Higher Education and Research, Wardha, Maharashtra, India; ^9^South Asia Infant Feeding Research Network (SAIFRN), Wardha, India; ^10^Department of Community Medicine, Jawaharlal Nehru Medical College and Faculty, School of Epidemiology and Public Health, Datta Meghe Institute of Higher Education and Research, Wardha, Maharashtra, India; ^11^NewGen IEDC, Department of Research and Development, Datta Meghe Institute of Higher Education and Research, Wardha, Maharashtra, India

**Keywords:** rapid-cycle, child development, self-reflection, intervention delivery, integrated program, monitoring and evaluation

## Abstract

**Background:**

Effective and real-time data analytics plays an essential role in understanding gaps and improving the quality and coverage of complex public health interventions. Studies of public health information systems identify problems with data quality, such as incomplete records and untimely reporting. Effective data collection and real-time analysis systems for rapid-cycle learning are necessary to monitor public health programs and take timely evidence-based decisions. Early childhood development (ECD) programs are very diverse. Rapid-cycle evaluation and learning (REAL) guides the implementation process of such complex interventions in real time. Stepping stones was one such early childhood development program implemented in Central India.

**Objective:**

The objective was to improve the delivery of complex, integrated public health interventions for early childhood development in remote areas of rural India.

**Methodology:**

The program was developed according to the principles of inclusion and community-centeredness, which can be tested quickly and iteratively. To enhance the decision-making process and improve delivery and coverage, the core team implemented an information system for rapid-cycle learning. We developed performance indicators and a performance measurement matrix after defining the specific needs. Following that, we trained staff to collect complete data using electronic data collection tools and transfer it the same day to the server for quality review and further analysis. A variety of data/information was triangulated to address the gaps in intervention delivery, and those decisions were subsequently implemented.

**Results:**

We observed that the quality of data collection improved, and errors were reduced by 50% in the third quarter. The quality of the narrative was also enhanced; it became more elaborate and reflective. Sharing their field output in meetings and improving the quality of the narrative enhanced the self-reflection skills of field staff and consequently improved the quality of the intervention delivery. Refresher training and mentoring by supervisors helped to improve the data quality over time.

**Conclusion:**

Rapid-cycle evaluation and learning (REAL) can be implemented in resource-limited settings to improve the quality and coverage of integrated intervention in early childhood. It nurtures a reinforcing ecosystem that integrates providers, community, and family perspectives and guides interactions among stakeholders by integrating data from all available sources.

## Introduction

Effective and real-time data analytics plays an essential role in understanding gaps and improving the quality and coverage of complex public health interventions. Studies of public health information systems identify problems with data quality, such as incomplete records and untimely reporting (1). Too often, healthcare information is disconnected and not readily accessible in a centralized, informed manner, significantly limiting the efforts to make informed decisions that improve the implementation of public health interventions (2). Effective data collection and real-time analysis systems for rapid-cycle learning are necessary to monitor public health programs and take timely evidence-based decisions (3–5).

Early childhood development (ECD) programs are very diverse and often build on a one-size-fits-all model guided by broad generic goals which are relatively ineffective (6). Families and communities benefit the most when ECD programs are adjusted to specific and contextual needs. A well-designed intervention strategy, proper staff recruitment, training, certification, and careful monitoring of service delivery over time contribute to the successful, quality implementation of these complex interventions in early childhood. Access to local information makes a key contribution to achieving these objectives.

The rapid-cycle evaluation and learning (REAL) approach guides the implementation process of such complex interventions in real time. Rapid-cycle learning requires an information system that pools scattered data and multiple sources of information on program implementation at one central location. The role of this central hub is to clean, compile, analyze, and share information with relevant stakeholders to guide collaborative interpretation and rapid decision-making. Information shared by a REAL approach should guide program managers in effective planning, training, implementation, and evaluation. This study shares examples of the role of information systems and rapid-cycle learning for timely decision-making. The objective was to improve the delivery of complex, integrated public health interventions for early childhood development in a remote area of rural India.

## Methodology

### The context and settings

We implemented the “Stepping Stones” program in underserved rural, remote central India to promote early child development (ECD). The project implementation site is one of the most underprivileged populations in the region, with an average annual per-capita income below the state average. The population faces the stresses of irregular income and food insecurity and poor housing. Traditional practices are widely prevalent in these regions, strongly influencing health-seeking behaviors and childcare practices. Parents with limited education and exposure to new knowledge and skills face the challenge of limited availability and accessibility to health, education, and social services to support them in being responsive parents. The “Stepping Stones” program involves multiple approaches aimed at promoting a nurturing environment in the early years of development (0–6 years) (7, 8). The program, aimed at shaping the interactions and child experiences, includes interventions to enhance caregivers' skills and competencies in child stimulation, care, and nutrition, and interventions to support government Anganwadi Centers in their delivery of child-centered, play-based early childhood education (ECE). In addition to these activities, we organized workshops on making low-cost toys, set up a nutritional demonstration center, promoted vegetable gardens, and conducted community awareness through group meetings (8, 9). In the early stages of implementation, the challenge of providing such a complex program, with the desired quality, in remote rural areas through community volunteers was noted. The core team established an information system for rapid-cycle learning to complement the decision-making process and improve the delivery and coverage of the intervention. A delivery system was developed that was adapted to the local needs based on building a relationship between families and community peer mentors, Balsakhi, who, in turn, was supported by the existing network of government Anganwadi workers.

While we used a cluster randomized trial to evaluate the effectiveness of the “Stepping Stone” program in promoting the development of children from rural India, the key process elements were evaluated through in-depth analysis and by developing innovative mixed methods (10).

### Ethics approval

The Institutional Ethics Committee approved the Stepping Stones Trial of the Datta Meghe Institute of Medical Sciences (Deemed to be University) *via* a letter with the reference number DMIMS (DU)/IEC/2014-15/1203 dated 31 March 2015. The preliminary trial was registered with International Standard Randomized Controlled Trials with the trial number: ISRCTN87426020. The nature and objective of the study were explained to the study participants and their families during counseling sessions. The researcher obtained written consent after assuring participants of the confidentiality of the data.

### Approach

**Figure 1:**
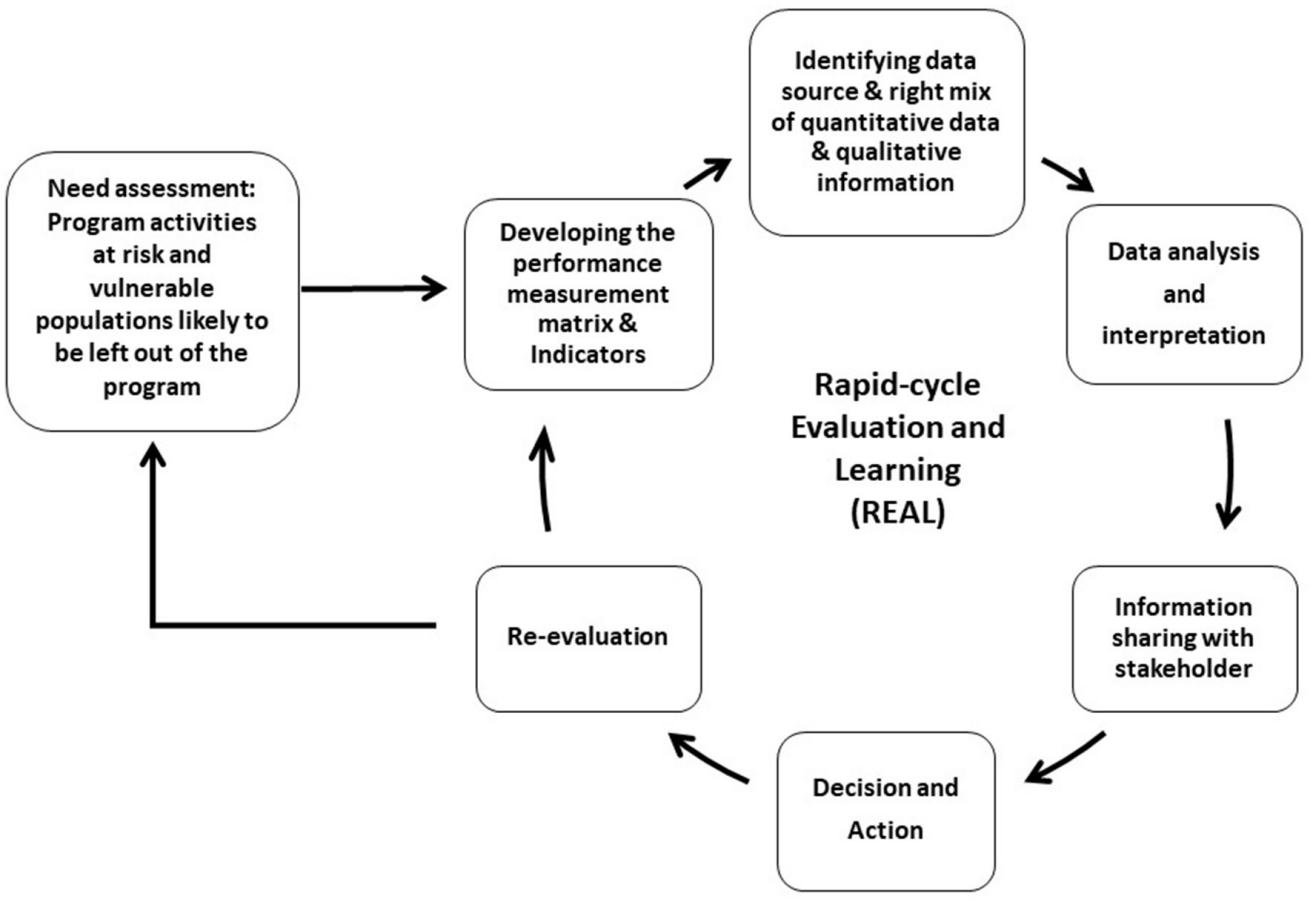
Overview of the rapid-cycle evaluation and learning.

We adopted principles of inclusion and community-centered that can quickly and iteratively test program implementation. We aimed to provide real-time information to program implementers and other stakeholders for the continuous improvement of program delivery. Figure 1 presents the overview of the proposed REAL approach. We expand upon the components in the following text.

### Needs assessment

The framing of the REAL system was guided by the need to be feasible, durable, and accountable, with system planning considering questions such as, what specific intermediate and primary outcomes will we hold ourselves accountable? How will we use the information? What should be measured and to what depth? How are we going to track progress, including when and how often data will be collected? Finally, what resources are needed to meet these objectives? The REAL framework was closely linked to the needs of the implementation process itself. This meant understanding population characteristics and dynamics and identifying those most at risk of adverse outcomes, the most vulnerable, and those most likely to be missed. In the formative phase, we completed two FGDs with community members and three key informant interviews, one with a service provider, one with a community member, and one with a government Anganwadi Worker (ECD staff); and we reflected on other information through a series of brainstorming sessions between core project staff.

### Performance measurement matrix and indicators

After defining the specific needs, we developed a performance measurement matrix and defined performance indicators. The primary purpose of the matrix was to help program implementers in comparing the intervention data on what happened to what was planned and to track the progress of the intervention.

### Identifying data source

The core team identified the blend of quantitative data and qualitative measures to capture. We also reviewed indicators used for global monitoring, and selected indicators that are locally relevant, feasibility of measurement, data availability, and relevant to stepping stones intervention (11). Subsequently, we trained staff to capture complete data on electronic data collection tools and transferred it to the server the same day to be available for quality review and further analysis. The next step was data analysis and interpretation.

### Data analysis and interpretation

In the primary trial, we aimed to detect the desired improvement of 0.3SD in development score in the intervention group, with 95% confidence and 80% power, a total sample size of 452 mother–child dyads. Based on the previous experience, we accounted for a 20% loss to follow-up in the sample size. Therefore, the final sample size is 542 from 21 clusters, with 271 in each group. However, from an ethical perspective, all eligible participants fulfilling the inclusion criteria from the intervention and control clusters were enrolled in the study. Moreover, we assessed 814 participants for eligibility and recruited 656 after meeting the eligibility criteria (326 from the intervention cluster and 330 from the control cluster). For this study, we considered all participants (326) from the intervention arm.

All variables were tested for normal distribution using a graphical method by plotting histograms. Continuous variables with normal distribution are expressed as mean ± standard deviation (S.D.). Categorical variables are expressed as numbers (percentage). Comparisons between independent groups were made using Student's *t*-test. Categorical data were compared with the chi-square test and Fischer's exact test was performed. All tests performed were two-sided. Single linear regression analysis was used to examine correlations between hemoglobin levels and continuous variables. Variables included in the multivariate model were fixed variables, which are known as confounders and risk factors. A *p*-value of < 0.05 was considered statistically significant.

**Figure 2:**
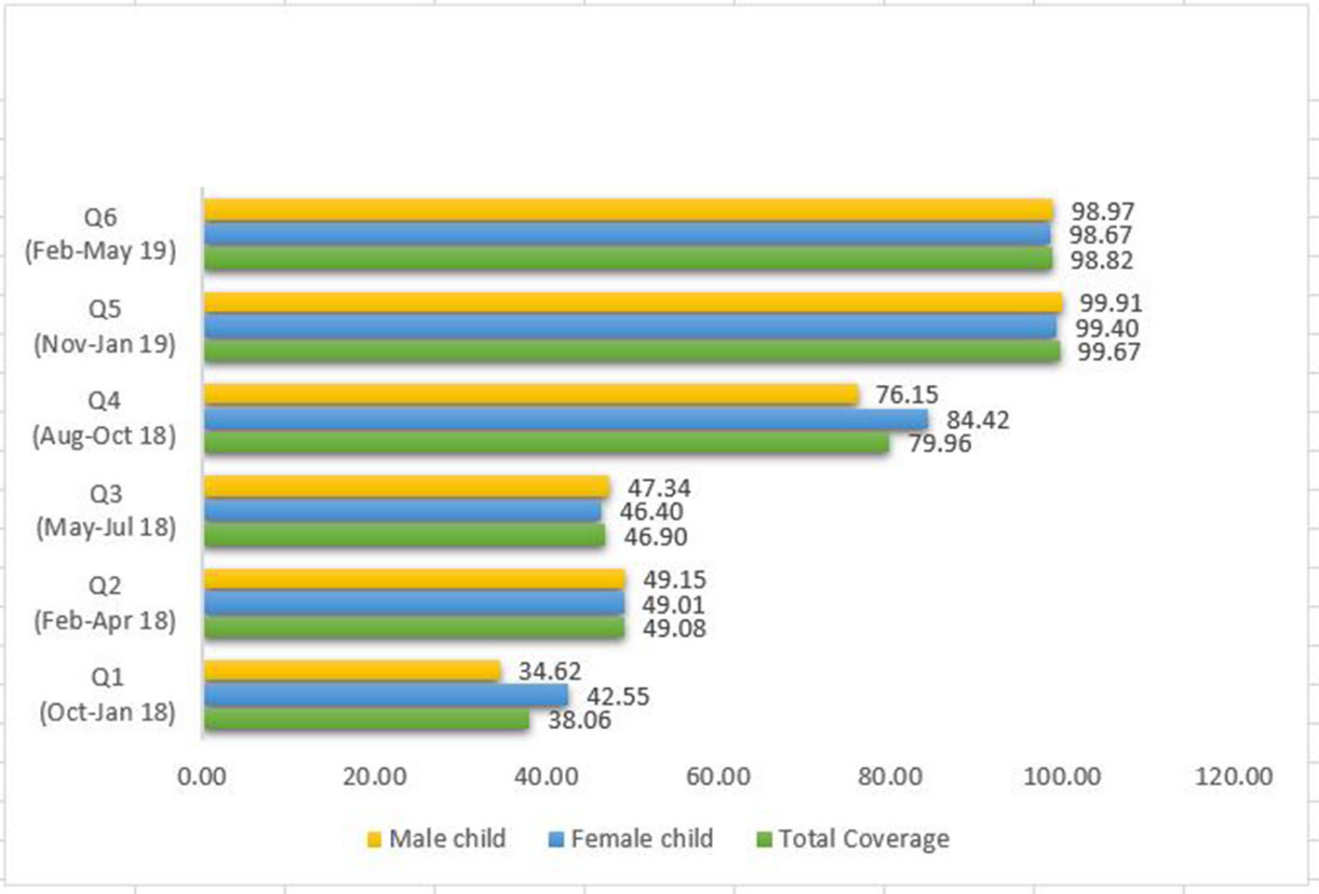
Percentage coverage with quarter review cycles.

We developed an electronic platform to collect, manage, and analyze data in real time. We used an Open Data Kit (ODK) that allows data collection using Android mobile devices. Figure 2 presents the actual flow of information from the field to a central server, as well as data handling and cleaning, analysis, and sharing. The project supervisor transferred all the data to the electronic device, weekly, preferably on Friday. The entire data were then pushed to the server on the same day. The data manager then retrieved all data on Saturday and ran the predefined codes to generate output related to the core process indicators. The server data were extracted and analyzed for predefined key indicators. We used STATA/MP Version 15 to generate the output for key process indicators and discussed it in stakeholder review meetings. A variety of data/information was triangulated—data captured by service providers, narratives from the field, supervisors' data and photographs captured by supervisors, and previous meeting notes were discussed to understand what is going on well, what components were challenging, and why as well as potential gaps in intervention delivery and possible decisions for addressing those gaps. The first cycle of the critical review was conducted 3 months after the intervention went live to make decisions to guide. Subsequently, it was done on monthly basis.

### Information sharing with stakeholders

The project team shared the status of the indicators and outcomes with all stakeholders and the community in a monthly/quarterly meeting. The purpose of these meetings was to review the actual status, track intervention progress, and make new decisions regarding program implementation if needed. In addition to quantitative data, qualitative information such as service delivery data, supervisory data, service providers' daily log, field photographs of home visits, and notes of the previous meetings are discussed. The service providers were asked to write down a brief narrative about the home visits as reflective learning tools. The qualitative information and quantitative data are triangulated to draw inferences about the service gaps, and decisions were taken in meetings itself to address those gaps. The frequency of analysis and sharing depends on the outcomes/indicators. For example, some of the target outcomes, such as child development domains, may occur in the later stage of the program to be examined in a rapid evaluation, whereas some intermediate outcomes such as home environment and caregiver–child interaction may start showing changes early and can be considered for the rapid evaluation.

### Collaborative decision-making

Based on the indicators/data review, project staff, community, and stakeholders collaboratively took the necessary decisions to address the gaps in the intervention delivery, and those decisions were subsequently implemented. Finally, in the next cycle, we re-evaluated the new decision implemented and any other new challenges in the intervention delivery. These cycles continued till the end of the intervention period, and the entire process was collaboratively conducted by the project team, community members, service providers, and other stakeholders.

## Results

### Needs assessment

Table 1 presents the characteristics of participants during enrollment. The needs assessment exercise identified the population subgroups most likely to be excluded from the program and the program components most difficult to deliver as those living on the periphery of the villages, specific cast groups, lower economic class, single parents, and families exposed to the consequences of alcohol abuse. These were prioritized in the performance measurement matrix. While each intervention component was not felt to be challenging to deliver; data analysis showed that the quality of delivery required improvement.

**Table 1:** Baseline characteristics of study participants in the intervention arm.

**Maternal characteristics**	**Intervention (*****n*** = **326)**	
	**No**	**%**
**Age in years;** Mean (SD)	23.79 (3.57)	
**Education**		
Illiterate	11	3.37
Primary (1–5)	12	3.68
Secondary (6–10)	156	47.85
Higher secondary	100	30.67
Graduate and more	47	14.42
**Pregnancy duration**		
1^st^ Trimester	68	20.86
2^nd^ Trimester	258	79.14
**Gravida**		
First	150	46.01
Second	145	44.48
Third	26	7.98
Fourth	4	1.23
Fifth	1	0.31
**Total of live children;** Mean (SD)	0.61 (0.68)	
**Anemia**		
No Anemia	82	29.82
Mild Anemia	96	34.91
Moderate Anemia	95	34.55
Severe Anemia	2	0.73
**Father's characteristics**		
**Age in years**; Mean (SD)	29.59 (3.63)	
**Education**		
Illiterate	13	3.99
Primary (1–5)	23	7.06
Secondary (6–10)	175	53.68
Higher Secondary	82	25.15
Graduate	33	10.12
**Household characteristics**		
**Caste category**		
Schedule caste	24	8.11
Schedule tribe	141	47.64
Backward classes	123	41.56
Open/General	8	2.70
**Wealth index**		
1^st^ Quintile	49	15.03
2^nd^ Quintile	58	17.79
3^rd^ Quintile	83	25.46
4^th^ Quintile	75	23.01
5^th^ Quintile	61	18.71
**Average family size**; mean (SD)	4.86 (1.91)	
**Below poverty line**	145	44.62

### Performance measurement matrix

Indicators to measure the quality-of-service delivery included the proportion of scheduled visits completed on time; the average duration of each visit; the number of home visits supervised by the Anganwadi workers or project supervisors; home visits that were more interactive and engaging. We also defined indicators to access overall intervention coverage, as well as coverage for villages, male and female children, vulnerable or most at-risk families, and the household wealth index. As some of the core outcomes of the program may take time to show changes, we also defined intermediate outcome indicators for tracking progress that include average weight gain during pregnancy; the woman who started breastfeeding within 1 h, birth weight of babies/low birth weight (< 2,500 g). Coverage was defined as the number of caregivers receiving parenting sessions through home visits (numerator) as planned compared to the total expected beneficiaries (denominator).

### In-depth reviews

In addition to a monthly review of core indicators, an in-depth review was conducted quarterly. The data/information from all sources, such as intervention data, field photographs, and supervisory data, were triangulated and presented in the quarterly meetings to identify intervention gaps and take an evidence-based decision. The details of indicators on the coverage and quality indicators are presented in Figure 2, Tables 2, 3.

**Table 2:** Intervention coverage in quarterly review cycles with wealth quantiles and caste categories.

**Quarters**		**(Oct–Jan 18)**	**(Feb–Apr18)**	**(May–Jul 18)**	**(Aug–Oct 18)**	**(Nov–Jan 19)**	**(Feb–May 19)**
**Wealth quantiles**							
Lowest quantile	Expected	337	293	325	343	318	203
	Reached No (%)	131 (38.87)	149 (50.85)	128 (39.38)	250 (72.88)	265 (83.33)	192 (94.58)
Second quantile	Expected	328	310	381	397	378	306
	Reached No (%)	123 (37.50)	195 (62.90)	179 (46.98)	345 (86.90)	362 (95.77)	299 (97.91)
Third quantile	Expected	395	428	536	554	531	454
	Reached No (%)	140 (35.44)	223 (52.10)	237 (44.22)	534 (96.39)	525 (98.87)	438 (96.48)
Fourth quantile	Expected	300	371	484	498	476	459
	Reached No (%)	111 (37.00)	182 (49.60)	180 (37.19)	405 (81.33)	437 (91.81)	452 (98.47)
Highest quantile	Expected	248	344	438	464	445	450
	Reached No (%)	107 (43.15)	225 (65.41)	189 (43.15)	388 (83.62)	441 (99.10)	446 (99.11)
**Caste category**							
Open/General	Expected	53	59	70	72	71	59
	Reached No (%)	28 (52.83)	31 (52.54)	32 (45.71)	58 (80.56)	62 (87.32)	55 (93.22)
Schedule cast	Expected	157	152	187	207	196	158
	Reached No (%)	63 (40.13)	114 (75.00)	86 (45.99)	182 (87.92)	185 (94.93)	150 (94.94)
Schedule tribes	Expected	826	857	1,032	1,064	989	829
	Reached No (%)	274 (33.17)	365 (42.59)	365 (35.37)	853 (80.17)	934 (94.44)	770 (92.88)
Other backward class	Expected	572	678	875	913	892	826
	Reached No (%)	252 (44.06)	399 (58.58)	492 (56.23)	841 (92.91)	885 (9.22)	818 (99.03)

**Table 3:** Indicators on intervention delivery by the service provider with the review quarters.

**Quarters**	**(Oct–Jan 18)**	**(Feb–Apr 18)**	**(May–Jul 18)**	**(Aug–Oct 18)**	**(Nov–Jan 19)**	**(Feb–May 19)**
The average duration of a home visit (minutes)	25.90	38.65	37.96	36.45	37.61	36.79
Home visit ≤ 15 min; No (%)	109 (18.73)	2 (0.24)	0	0	0	0
Home visit 16–30 min; No (%)	251 (43.13)	106 (12.79)	106 (10.44)	194 (10.75)	205 (9.32)	207 (11.19)
Home visit 31–45 min; No (%)	154 (26.46)	473 (57.06)	669 (65.91)	1,095 (60.70)	1,356 (61.66)	1,374 74.27)
Home visit > 45 min; No (%)	68 (11.68)	248 (29.92)	240 (23.65)	515 (28.55)	638 (29.01)	269 (14.54)
Family centered/interactive; No (%)	139 (23.88)	458 (55.25)	755 (74.38)	1,599 (88.64)	1,971 (89.63)	1,654 (89.41)
Male participations; No (%)	25 (4.30)	42 (5.07)	59 (5.81)	301 (16.69)	718 (32.65)	579 (31.30)

In the first 3 months of intervention, we observed that the service provider was able to reach only one-third of the expected beneficiaries. Timely intervention increased coverage from 32 to 98% in the first quarter of intervention. A proportion of beneficiaries reached through home visits were affected during the summer and early rainy season period or the third quarter. A similar trend was observed in the wealth quantiles of male and female children. The coverage of intervention for male and female children was not significantly different (*p* > 0.05) (Figure 2).

We assessed the session delivery with minimum desirable standards. Table 2 presents the details of core indicators for the session quality. After the first review, we observed a need for the intervention to improve the session quality in almost all aspects. The overall average duration was 35.59 min. The proportion of home visits lasting between 30 and 45 min increased from the first to the sixth quarter, and the highest percentage (74.27%) was reported in the sixth quarter. Participatory interaction was defined as family members proactively participating in discussion and posing at least five questions or queries to service providers visiting the household. The percentage of home visits with participative interaction went on increasing from the first quarter to the sixth quarter and the highest percentage of visits with participative interaction (89.63%) was reported in the fifth quarter. Participation of male participants in home visits was limited in the beginning (4.3%) but increased to 32.65% in the last quarter. In the first quarter, on average, there were 270 data errors per month, which had been reduced to 140 by the end of the third quarter. The most common errors in data collection were missing data, out-of-range values, and digit preferences.

## Discussion

The rapid-cycle evaluation and learning approach is used for more than just describing the status of interventions/programs and providing trial implementers with evidence on what to do next. Integrated community-based interventions require new ways to collect and analyze data in rapid cycles. The program managers can link the quantitative data with non-traditional sources such as narratives from the field, photographs, and meeting notes to generate new insights for evidence-based decision-making at the right time (11–13). Traditionally, coverage and quality seem like a trade-off (11). Our approach effectively improved the coverage without compromising the quality of service delivered in spite of our intervention being implemented in the remote and rural setup. The REAL approach provides continuous learning opportunities to identify and analyze gaps and takes timely evidence-informed decisions to improve the implementation of integrated intervention delivered in early childhood. The FAMI program in Colombia demonstrated similarly how the multimethod approaches could assess the quality and inform the existing programs to enhance their design and implementation in a research context (14).

We observed gaps in the first quarterly review, which made us reflect on our entire intervention implementation design and make evidence-based decisions to address the implementation gaps. In discussions with key stakeholders, we dive deeper into the reasons for the intervention's failure. In addition to temporary migration (nearly one-fourth of the population from the study villages), a closer analysis of data, narratives, and photographs suggest that a relatively complex nature of intervention, a lack of community engagement, and a lack of trust between community and service providers leads to low uptake of the intervention. Our approach to triangulating quantitative intervention data with the supervisor's checklist, field photographs, and meeting notes reveals that the service providers/field volunteers needed continued ongoing coaching, mentoring support, and supportive supervision.

To deliver the sessions effectively, the service providers/Balsakhi should have an engaging interaction with the caregiver for not <30 min. Even though the field service providers were trained and certified, the quality of home visit sessions was more directive, focused on information sharing and advising, less interactive, and not adaptive to the needs of mothers/caregivers. Service providers/Balsakhi were reading the manual to the mother point by point, which was, in fact, developed to guide them to deliver the session effectively. The field photographs and supervisory feedback in the first quarter showed overall low confidence and motivation of Balsakhi—community peer mentor, in delivering sessions and needed mentoring support. This leads to low interest among caregivers, leading to low coverage. Moreover, the implementation data were inconsistent, had missing values, out-of-range values, a preference for digits, and were not in a uniform format. This posed challenges for data merging and deriving the desired outputs.

The core project staff conducted a series of interactive discussions with key stakeholders—community, service providers, and government ICDS staff, which led us to take critical decisions, which are presented in Box 1.

Box 1 **Key decisions taken at various stages of the implementation cycle to improve the quality and coverage of intervention.**

1. To generate visit lists daily for all the service providers to help them schedule their visits and identify the coverage gaps daily, and to reach out to missed households within a week.
2. We appointed field supervisors who will be accessible and responsive to the needs of service providers at the village level. Field supervisors received a monthly remuneration and provided support to 8–10 service providers. We trained, certified these supervisors in ECD, supportive supervision, and mentoring. The implementation team developed a supervisor guidebook and checklist to facilitate a supportive leadership. Supervisors were supposed to accompany service providers in at least 50% of home visits and provide feedback to the field service providers and on-sight handholding/training if needed.
3. Retraining the field staff and service providers to improve data quality, reduce data errors, and write a weekly narrative to reflect on their performance.
4. Providing monthly feedback to individual service providers, sharing data gaps, identifying the specific issues, and addressing those.
5. Recognizing the contribution of service providers/Balsakhi to boost their confidence and motivation, which is critical for success.
6. Recruitment of male service providers and use of Photostory approach to promote male to engagement childcare activities.

On implementing the decisions to improve program performance, we observed that it took a bit for coverage to improve; however, the coverage was subsequently increased and sustained after the fifth quarter. The open caste category coverage was higher than the other categories in the first review, which were almost equal by the end of the sixth quarter through the targeted effort. The daily visit list has helped the service providers/Balsakhi to plan and make preparation for their sessions in advance. This reduces the chances of missed visits. The feedback loop and recognition of the work enhance the confidence and motivation of the village-level service providers. The supportive supervision and refresher training empower and equip the community peer mentor and other project staff to deliver the session effectively and timely. This was evident after the comparison of indicators over different review periods. The home visit sessions were delivered through an interactive process, as family-focused and not just mother-centered. The content was adapted to the felt need of the family.

Traditionally, childcare is viewed as a women's responsibility in households. However, we do have some success in promoting male participation in home visit sessions. Recruitment of male service providers, creating awareness at the community level, and the use of the Photostory approach (10) help us curate a social space for male participants to engage in household childcare activities.

The strength of our approach was a collaborative and inclusive process, which uses the strengths of stakeholders at all levels, including the community members. Unlike other studies that use real-time data analysis (15, 16), our approach was guided by the need assessment undertaken before the intervention rollout. In our approach, we identified the specific vulnerable population at risk of dropping out from the intervention and the intervention components challenging to implement through initial series of discussions with key stakeholders. This provides an opportunity to develop a matrix for capturing and tracking these indicators right from day one of intervention. However, one of the challenges of the approach is that the service providers and field staff collected the primary data; therefore, it was critical to collect high-fidelity data. Refresher training and mentoring by supervisors helped to improve the data quality over time. We observed that the quality of data collection by field staff improved, and errors were reduced to almost half. The quality of the narrative was also enhanced; it became elaborate and reflective. Earlier, it was just a bullet point of the services delivered by them. Sharing their field output in meetings and improving the quality of narrative enhanced self-reflection skills, which improved the quality of intervention delivery.

Our approach of using photographs and narratives brought change beyond technical knowledge and skills. It recognizes the importance of human and social values for staff delivering the intervention. Sharing the data with the service providers helped to reflect on their own performance, identify gaps, and make collective decisions. Feedback sharing in meetings always began with appreciative comments to help service providers understand the significance of their work for the success of the project and to bring positive changes within the community as well. This brings a sense of ownership for the intervention in the community and Balsakhi/service providers at the village level.

The approach has shown improvement in coverage and the quality of intervention for early childhood development. However, due to the lack of a control group, it limits our ability to attribute changes only to the REAL framework. Even though our approach was structured, it evolved further with our learnings throughout the course of the intervention. However, the triangulation of intervention data with narratives/stories from the field and photostories revealed that our approach significantly contributed to improving program implementation. The possibility of underestimating the coverage cannot be ruled out, as the 34 beneficiaries, who dropped out from the intervention were not excluded from the denominator, due to the lack of data on a specific time point at the drop-out. We failed to systematically capture this data. However, despite this limitation, our approach provides an important insight for the program manager to make an evidence-based decision to enhance the quality of intervention and to reach out to those who have the greatest possibility to be missed out on the program.

## Conclusion

Our approach of rapid-cycle evaluation and learning can be implemented in resource-limited settings to improve the quality and coverage of integrated intervention in early childhood. It nurtures a reinforcing ecosystem that integrates providers, community, and family perspectives and guides interactions among stakeholders by integrating data from all available sources. By analyzing and triangulating data in a variety of forms, we were able to overcome the limitations of each data form and provide a more holistic understanding of the issues to make informed high-impact decisions. This approach enhances the confidence and engagement of frontline service providers and brings credibility to their work. Our story highlights the need for a REAL framework to guide the fidelity of complex-integrated intervention delivery in early childhood.

## Data availability statement

The original contributions presented in the study are included in the article/supplementary material, further inquiries can be directed to the corresponding author.

## Ethics statement

The studies involving human participants were reviewed and the Institutional Ethics Committee approved the Stepping Stones Trial of the Datta Meghe Institute of Medical Sciences (Deemed to be University) *via* a letter with reference number DMIMS (DU)/IEC/2014-15/1203 dated 31.03.2015. Written informed consent to participate in this study was provided by the participants' legal guardian/next of kin.

## Author contributions

AG, MP, PH, and ZQ contributed to the conception and design of the study. AG wrote the first draft of the manuscript. ST, MP, SC, SG, and MK wrote the sections of the manuscript. RU and AP organized the database and data collection. PH reviewed and edited the manuscript. ST and AG performed the statistical analysis, data interpretation, data visualization, and figures. All authors contributed to the manuscript revision, read, and approved the submitted version.
